# Comorbid cerebrovascular and neurodegenerative burden in mild behavioural impairment and their impact on clinical trajectory

**DOI:** 10.1017/neu.2025.8

**Published:** 2025-03-13

**Authors:** Cheuk Ni Kan, Saima Hilal, Xin Xu, Narayanaswamy Venketasubramanian, Christopher Chen, Chin Hong Tan

**Affiliations:** 1 Memory Aging & Cognition Centre, Department of Pharmacology, Yong Loo Lin School of Medicine, National University of Singapore, Singapore; 2 Psychology, School of Social Sciences, Nanyang Technological University, Singapore; 3 Saw Swee Hock School of Public Health, National University of Singapore and National University Health System, Singapore; 4 School of Public Health and the Second Affiliated Hospital, Zhejiang University School of Medicine, China; 5 Raffles Neuroscience Centre, Raffles Hospital, Singapore; 6 Department of Psychological Medicine, Yong Loo Lin School of Medicine, National University of Singapore, Singapore; 7 Lee Kong Chian School of Medicine, Nanyang Technological University, Singapore

**Keywords:** Mild behavioural impairment, Neuropsychiatric symptoms, Magnetic resonance imaging, Cerebrovascular disease, Alzheimer’s disease, Mixed dementia

## Abstract

**Aim:**

Mild behavioural impairment (MBI) is a neurobehavioral prodrome to dementia with multiple phenotypic characteristics. To investigate the complex neurobiological substrate underlying MBI, we evaluated its association with a composite magnetic resonance imaging (MRI)-based measure of concomitant cerebrovascular disease (CeVD) and neurodegeneration; and the interaction effects of MBI and MRI scores on cognitive and clinical trajectory.

**Methods:**

253 dementia-free participants (mean age = 71.9, follow-up period = 49.89 months) from 2 memory clinics were included in this study. 37 (14.6%) participants met clinical diagnostic criteria for MBI, ascertained by repeated neuropsychiatric inventory assessments. MRI scores were computed using a validated weighted sum of white matter hyperintensities volume, presence of infarct, hippocampal volume, and cortical thickness of known Alzheimer’s disease-associated regions. Clinical and cognitive outcomes were evaluated annually using the Clinical Dementia Rating sum-of-boxes (CDR-SB) and standardised global cognitive scores of a comprehensive neuropsychological battery respectively.

**Results:**

Lower MRI scores, indicating greater burden of comorbid CeVD and neurodegeneration, yielded a 3.8-fold likelihood of MBI compared to 1.5-fold with larger WMH volume or lower cortical thickness individually. Interaction analyses showed that MBI participants with low MRI scores had greater increase in CDR-SB (B = 0.05, SE = 0.01, *p* < 0.001) over time. All models involving the composite MRI measure yielded a better fit compared to reduced models with either pathology alone.

**Conclusion:**

MBI is associated with a composite MRI measure that reflects mixed pathologies of dementia and their co-evaluation may improve risk profiling and identification of memory clinic patients without dementia who are at the highest risk of experiencing clinical decline.

## Graphical abstract

**Figure:**
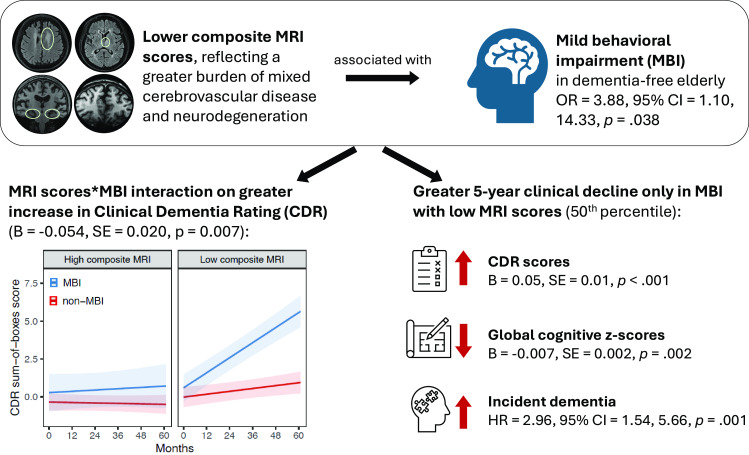

Significant outcomes

- Greater WMH volume and lower cortical thickness of known AD-related regions were associated with MBI.
- Composite mixed cerebrovascular disease and brain atrophy significantly increases MBI likelihood.
- Burden of mixed vascular and degenerative pathologies moderates the poor prognosis of dementia-free elderly with MBI.

Limitations

- The use of longitudinal NPI data to diagnose MBI may have underrepresented the prevalence of MBI.
- Study was conducted in a memory clinic cohort and may not be generalisable to other populations with lower pathological burden.
- Findings were restricted to MRI biomarkers of AD and cerebrovascular disease, which may be less economical than plasma biomarkers.

Highlights

- A composite MRI burden of cerebrovascular disease and brain atrophy significantly increased MBI likelihood compared to individual MRI biomarkers.
- Individuals with MBI and high MRI burden of mixed pathologies showed the greatest cognitive decline and dementia risk over 5 years.
- Co-evaluation of MBI and composite MRI burden may improve identification of memory clinic patients with the highest risk of clinical decline.

## Introduction

Neuropsychiatric disturbances are changes in mood and behaviour that are frequently associated with neurodegenerative diseases. While they are most commonly observed in people with dementia, recent evidence suggests that neuropsychiatric disturbances may develop years before disease onset (Wise *et al*., 2019; Liew, 2020), possibly reflecting an early behavioural manifestation of dementia due to underlying pathophysiological abnormalities. The role of neuropsychiatric disturbances as a neurobehavioral prodrome to all-cause dementia was further emphasised with the proposed diagnostic criteria for mild behavioural impairment (MBI) (Ismail *et al*., 2016). MBI, characterised by late-life persistent neuropsychiatric disturbances in the absence of dementia or major psychiatric disorders, has been demonstrated to be a robust syndrome in predicting faster cognitive decline and poorer prognosis (Creese *et al*., 2019; Rouse *et al*., 2020; Creese and Ismail, 2022; Kan *et al*., 2022a).

Recent magnetic resonance imaging (MRI) studies have uncovered significant structural brain differences associated with MBI. Greater cerebral atrophy of Alzheimer’s disease (AD)-associated regions, such as the entorhinal cortex, hippocampus, temporal gyrus, precentral gyrus, and middle frontal gyrus, have been implicated in MBI (Matuskova *et al*., 2021; Shu *et al*., 2021). Individuals with MBI were also found to have greater burden of white matter hyperintensities (WMH) (Miao *et al.,* 2021)^26^, another major cerebrovascular contributor to cognitive ageing and dementia (Wardlaw *et al*., 2013; Jellinger and Attems, 2015). However, some studies have also reported no association of brain atrophy or cerebrovascular disease (CeVD) with MBI (Lussier *et al*., 2020; Stella *et al*., 2022). The presence of these mixed MRI findings suggests that MBI may be a multifactorial syndrome influenced by both cerebrovascular and neurodegenerative pathologies.

In the investigation of disorders such as MBI with multiple phenotypic characteristics, capturing complex and heterogeneous pathophysiologic burden by combining biomarkers of underlying mechanisms may provide more statistical power than examining each MRI phenotype individually (Brickman *et al*., 2018; Wang *et al*., 2018; Xu *et al*., 2018; Jokinen *et al*., 2020). This study utilises a validated weighted composite MRI measure to quantify the combined influence of CeVD (WMH and infarcts) and neurodegeneration (hippocampus volume and cortical thickness of AD-associated regions) (Brickman *et al*., 2018). This composite MRI measure of mixed cerebrovascular and neurodegenerative pathologies was shown to be correlated with other AD biomarkers such as amyloid and tau (Brickman *et al*., 2018), and was better associated with multi-domain cognitive impairment (Tan *et al*., 2020) and neuropsychiatric syndromes (Kan *et al*., 2022b) than either pathology alone. Studying the relationship of a combination of multiple MRI phenotypes in conjunction with MBI likely captures a more comprehensive risk profile for predicting cognitive decline that is particularly crucial in the pre-dementia stage.

In this longitudinal memory clinic study, we evaluated the association of the quantitative MRI measure of comorbid CeVD and neurodegeneration with MBI status, and their interactive effects on clinical trajectories in dementia-free older adults. We hypothesised that a greater burden of comorbid CeVD and neurodegeneration would be associated with a higher likelihood of having MBI; and that the presence of MBI in conjunction with greater comorbid CeVD and neurodegeneration would be associated with faster cognitive and functional decline.

## Methods

### Study cohort

This study is part of an ongoing prospective cohort study that recruited participants from two memory clinics (National University Hospital and St Luke’s Hospital) in Singapore. Participants who were aged 50 years and above, had sufficient language skills for neuropsychological evaluations, and fulfilled clinical diagnostic criteria were enrolled into the study. Exclusion criteria included cognitive decline due to non-neurodegenerative or non-vascular causes (e.g. traumatic brain injury, multiple sclerosis, tumour, epilepsy, systemic disease) and major psychiatric disorders or substance abuse disorder as diagnosed according to the Diagnostic and Statistical Manual of Mental Disorders, Fourth Edition (DSM-IV) criteria.

All study participants underwent physical and clinical examinations, comprehensive neuropsychological evaluation, neuropsychiatric assessment, and brain MRI. Diagnosis was made at consensus meetings by neurologists and neuropsychologists after reviewing details of the brain scans and neuropsychological assessments. Participants without objective impairment on a locally validated standardised neuropsychological test battery (Narasimhalu *et al*., 2011) were diagnosed as ‘no cognitive impairment’; participants with impairment on at least one cognitive domain on the neuropsychological battery but remained functionally independent, hence not meeting DSM-IV criteria for dementia, were diagnosed with ‘cognitive impairment no dementia’; and participants with objective cognitive impairment and functionally loss were diagnosed with dementia following DSM-IV criteria.

### Standard protocol approvals, registrations, and patient consents

Ethics approval for the study was obtained from the National Healthcare Group Domain-specific Review Board (2015/00406-AMD0012). Written informed consent was obtained in the preferred language of all participants prior to enrolment according to the Declaration of Helsinki.

### MBI ascertainment

According to the International Society to Advance Alzheimer’s Research and Treatment-Alzheimer’s Association framework, MBI is characterised by changes in mood, perception, or behaviour that were later-life in onset, persistent at least intermittently for six months, and not caused by dementia or major psychiatric disorders (Ismail *et al*., 2016). The presence of behavioural and psychological symptoms was evaluated using the informant-based neuropsychiatric inventory (NPI) (Cummings, 1997), administered as a structured interview by trained neuropsychologist. As the NPI has a symptom reference period of one month, NPI data at both baseline and 1 year follow-up visits were utilised to assess the symptom persistence criterion of MBI, such that the presence of at least one symptom consecutively at baseline and 1 year follow-up was classified as MBI (Ismail *et al*., 2021; Kan *et al*., 2022a).

### Neuropsychological evaluation and dementia diagnosis

Cognitive performance was assessed annually for up to five years using a locally validated comprehensive neuropsychological test battery that comprises the Hopkins Verbal Learning Test (Brandt, 1991) Rey-Osterrieth Complex Figure Test (RCFT) – immediate and delayed recall and recognition, Colour Trails Tests 1 and 2 (D’Elia *et al*., 1996), Digit Span Test (Wechsler, 2009), Modified Boston Naming Test (Mack *et al*., 1992), Animal Fluency, Symbol-Digit Modalities Test (Smith, 1973), and RCFT-copy. All raw scores were standardised in the current sample to compute global composite z-scores indicative of global cognitive function. Clinical decline was evaluated annually using the Clinical Dementia Rating (CDR) Scale (Morris, 1993) and the CDR sum-of-boxes (CDR-SB) scores were tabulated for analysis. Dementia was diagnosed according to the DSM-IV criteria at consensus meetings after reviewing details of the clinical, neuropsychological, and neuroimaging examinations.

### Neuroimaging

#### MRI acquisition

MRI scans (3T MAGNETOM Trio™, A Tim® System; Siemens, Germany) were performed at the Centre for Translational Magnetic Resonance Research, National University of Singapore. High-resolution T1-weighted structural MRI was performed using a magnetisation-prepared rapid gradient echo sequence with 192 continuous sagittal slices, repetition time (TR) = 2300ms, echo time (TE) = 1.9 ms, inversion time (TI) = 900 ms, flip angle = 9 DA; and field of view (FOV) = 256 × 256 mm^2^. T2-weighted MRI was performed with 48 axial slices, voxel size = 1 × 1 × 3 mm^3^, TR = 2600ms, TE = 99 ms, flip angle = 150^°^, and FOV = 232 × 256 mm^2^. The T2-Fluid-attenuated inversion recovery (FLAIR) sequence was acquired with 48 axial slices, voxel size = 1 × 1 × 3 mm^3^, TR = 9000 ms, TE = 82 ms, TI = 2500ms, flip angle = 180^°^, and FOV = 232 × 256 mm^2^.

#### MRI processing

The presence of infarcts was visually graded based on the Standards for Reporting Vascular changes on Neuroimaging criteria (Wardlaw *et al*., 2013; Hilal *et al*., 2015). Infarcts are characterised as round or ovoid lesions in the subcortical regions, 3 to 15 mm in diameter, with a high signal on T2-weighted images and a hyperintense rim surrounding a centre following the cerebrospinal fluid intensity, and a low signal on FLAIR and T1-weighted images (Wardlaw *et al*., 2013). Volumetric WMH were quantified using an automated segmentation pipeline, where the T2-FLAIR images were first registered to the participants’ corresponding T1 images via linear registration before spatial normalisation using the Montreal Neurological Institute (MNI)-152 brain template. Structural T1-weighted images were processed using automated procedures in FreeSurfer 5.1 (https://surfer.nmr.mgh.harvard.edu/). Hippocampal volume was derived from the subcortical segmentation pipeline and cortical thickness of AD-associated regions (i.e. entorhinal cortex, para-hippocampus, inferior parietal, pars opercularis, pars orbitalis, pars triangularis, inferior temporal, temporal pole, precuneus, supramarginal gyrus, superior parietal, and superior frontal lobe) was derived from the cortical parcellation pipeline based on the Desikan-Killiany atlas (Desikan *et al*., 2006).

#### Quantification of comorbid cerebrovascular and neurodegenerative burden

The quantitative MRI indicator of comorbid CeVD and neurodegeneration was calculated as the weighted sum of the individual biomarkers of CeVD and neurodegeneration using a pre-established equation: (−0.088)*log WMH volume + (−0.045)*presence of infarct + (0.00027)*hippocampal volume + (1.03)*cortical thickness of known AD-associated regions (Brickman *et al*., 2018). The weights for the quantitative MRI indicator were previously derived from the associations of each biomarker of CeVD and neurodegeneration with episodic memory performance, which were subsequently validated in an independent sample (Brickman *et al*., 2018) and associated with clinical diagnosis and multi-domain cognitive decline in multiethnic Asians (Tan *et al*., 2020). Lower quantitative MRI scores indicate a greater burden of comorbid CeVD and neurodegeneration.

### Statistical analyses

First, we conducted logistic regression to evaluate the association of quantitative MRI scores with presence of MBI, adjusted for age, sex, education, clinical diagnosis, and total intracranial volume (TIV). We also examined whether individual MRI biomarkers of WMH volume (log-transformed), presence of infarct, hippocampal volume, and cortical thickness were associated with presence of MBI using logistic regression, adjusted for the same covariates. Second, we used linear regression to evaluate the associations of quantitative MRI scores and MBI diagnosis with CDR-SB and global cognitive z-scores at baseline, adjusted for age, sex, education, clinical diagnosis, and TIV. Interactions between quantitative MRI scores and MBI on CDR-SB and cognitive performance were further tested. Next, we examined the effects of quantitative MRI scores and MBI on longitudinal changes in CDR-SB and global cognitive z-scores using linear mixed models, adjusted for age, sex, education, clinical diagnosis, TIV, and all base terms interactions with time (mean follow-up period = 49.89 ± 16.53 months). Quantitative MRI scores, MBI, and their interaction were entered as fixed factors, and participants as random factor.

To determine if the quantitative MRI model resulted in a better fit compared to the non-nested models with only the unweighted CeVD biomarkers (WMH volume + infarct) or only neurodegeneration biomarkers (hippocampal volume + cortical thickness), we compared the Bayesian information criterion (BIC) differences for associations with significant effects. The Bayesian approach to model selection allows direct comparison of non-nested models, accounting for model uncertainty and avoiding the difficulties of standard model selection procedures (Raftery, 1995). This analytic approach has also been implemented in prior studies (Tan *et al*., 2020; Kan *et al*., 2022b). The BIC was used to guide the iterative model selection process, where a BIC difference of >10, 6–10, and 2–6 are equivalent to a Bayes factor of >150 (very strong evidence), 20–150 (strong), and 3–20 (positive) respectively (Raftery, 1995). Lastly, we used Cox proportional hazards models to evaluate the effects of quantitative MRI scores, MBI, and their interaction on progression to dementia, with time to dementia onset, adjusted for age, sex, education, clinical diagnosis, and TIV at baseline.

## RESULTS

### Cohort characteristics

A total of 407 dementia-free participants were enrolled between August 2010 and October 2020. Participants with incomplete MRI data for processing (N = 66) and neuropsychiatric data for MBI diagnosis (N = 88) were excluded, leaving a total of 253 dementia-free participants for analysis. There were no significant differences in demographics or cognitive status between included and excluded participants (all *p’s* >0.05). Amongst the 253 dementia-free participants included, 14.6% met clinical diagnostic criteria for MBI and 85.4% were non-MBI. Compared to non-MBI participants, participants with MBI scored lower on the Mini-Mental State Examination, had greater WMH volume, lower cortical thickness, and lower quantitative MRI scores (all *p’s* <0.05), indicating a greater burden of comorbid CeVD and neurodegeneration (Table 1).

**Table 1.** Baseline characteristics of study participants

	Total	MBI	Non-MBI	
	***N* = 253**	***N* = 37 (14.6%)**	***N* = 216 (85.4%)**	***p***
***Demographics***				
Age, M (SD)	71.9 (7.7)	73.2 (8.0)	72.2 (7.7)	0.236
Sex (female), n (%)	131 (51.8)	18 (48.6)	113 (52.3)	0.680
Education, M (SD)	8.1 (4.9)	8.3 (5.7)	8.0 (4.8)	0.751
MMSE score, M (SD)	24.8 (3.5)	**23.5 (3.2)**	**25.0 (3.5)**	**0.015**
Clinical diagnosis, n (%)				
No cognitive impairment	77 (30.4)	7 (18.9)	70 (32.4)	
Cognitive impairment no dementia	176 (69.6)	30 (81.1)	146 (67.6)	
***MRI characteristics***				
WMH volume (mL), median (IQR)	7.10 (12.0)	**12.40 (17.0)**	**6.71 (11.0)**	**0.008**
Presence of infarct, n (%)	63 (24.9)	10 (27.0)	53 (24.5)	0.746
Hippocampal volume (mm^3^), M (SD)	7046.87 (913.49)	6833.74 (807.62)	7083.38 (927.20)	0.125
Cortical thickness (mm), M (SD)	2.50 (0.12)	**2.46 (0.11)**	**2.51 (0.11)**	**0.014**
Quantitative MRI score, M (SD)	4.29 (0.35)	**4.15 (0.29)**	**4.31 (0.35)**	**0.009**

Abbreviations: MBI, mild behavioural impairment; MMSE, Mini-Mental State Examination; WMH, white matter hyperintensities.

### Association of quantitative MRI scores with MBI

Lower quantitative MRI scores, indicating greater burden of comorbid CeVD and neurodegeneration, were associated with a higher likelihood of MBI (OR = 3.88, 95% CI = 1.10, 14.33, *p* = 0.038). Among the individual MRI biomarkers, larger WMH volume (OR = 1.48, 95% CI = 1.06, 2.06, *p* = 0.018) and smaller cortical thickness (OR = 1.53, 95% CI = 1.00, 2.34, *p* = 0.048) were associated with MBI. The presence of infarct (OR = 1.02, 95% CI = 0.43, 2.30, *p* = 0.947) and hippocampal volume (OR = 1.23, 95% CI = 0.81, 1.87, *p* = 0.333) were not associated with MBI. However, all individual MRI biomarkers were not significant after adjustment for multiple comparisons (false discovery rate > 0.05 for four comparisons).

### Quantitative MRI scores, MBI, and clinical trajectory

**Figure 1.**
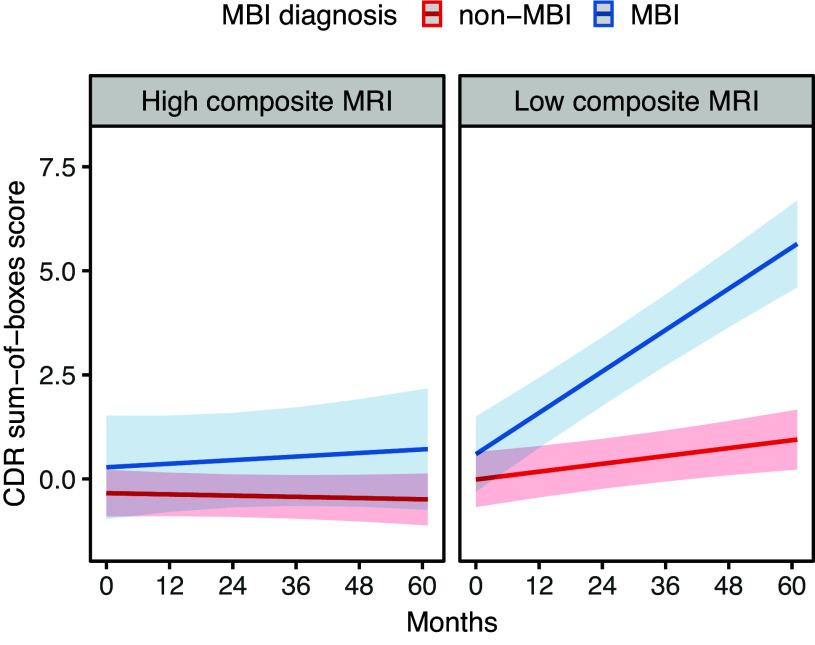
Change in CDR sum-of-boxes in MBI (blue) and non-MBI (red) participants, stratified by low/high quantitative MRI scores (50^th^ percentile). Stratified linear mixed models, adjusted for age, sex, education, clinical diagnosis, TIV, MBI status, time, and all base terms interactions with time showed greater increase in CDR sum-of-boxes in MBI participants with low (B = 0.05, SE = 0.01, *p* < 0.001) but not high (B = 0.002, SE = 0.008, *p* = 0.766) quantitative MRI scores.

At baseline, participants with MBI had higher CDR-SB (B = 0.34, SE = 0.15, *p* = 0.023), independent of quantitative MRI scores. No statistically significant interaction between quantitative MRI scores and MBI on CDR-SB was detected at baseline (B = 0.23, SE = 0.49, *p* = 0.640). Longitudinal analyses showed that MBI participants had greater increase in the CDR-SB over time (B = 0.049, SE = 0.007, *p* < 0.001), indicating greater clinical decline, that was independent of quantitative MRI scores. Quantitative MRI scores and MBI also showed significant interaction effect on CDR-SB rate of change (B = −0.054, SE = 0.020, *p* = 0.007), where MBI participants with low quantitative MRI scores showed the greatest increase in CDR-SB (B = 0.05, SE = 0.01, *p* < 0.001) (Fig. 1).

### Quantitative MRI scores, MBI, and cognitive trajectory

**Figure 2.**
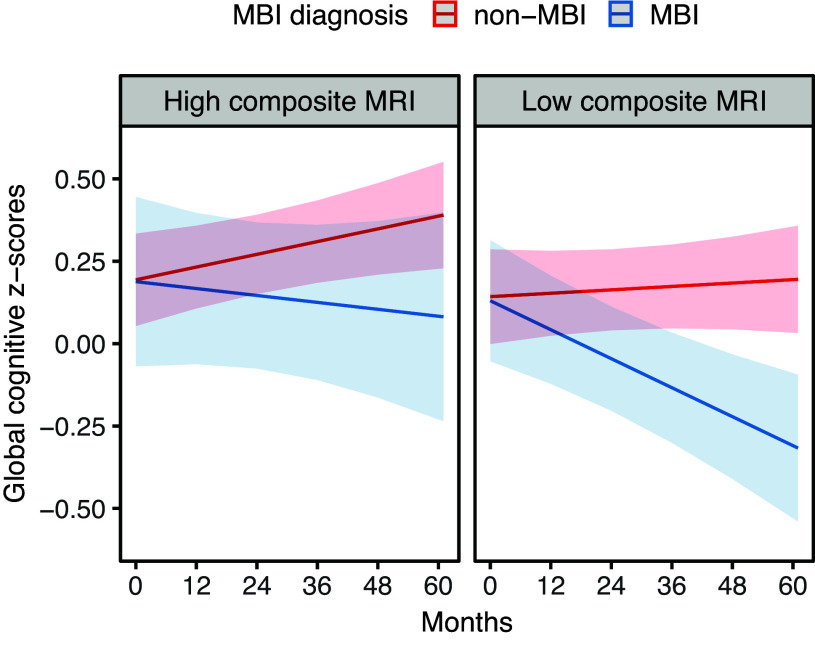
Change in global cognition in MBI (blue) and non-MBI (red) participants, stratified by low/high quantitative MRI scores (50^th^ percentile). Stratified linear mixed models, adjusted for age, sex, education, clinical diagnosis, TIV, MBI status, time, and all base terms interactions with time showed greater cognitive decline in MBI participants with low (B = −0.007, SE = 0.002, *p* = 0.002) but not high (B = −0.003, SE = 0.003, *p* = 0.265) quantitative MRI scores.

At baseline, the presence of MBI was associated with lower global cognitive z-scores (B = −0.27, SE = 0.12, *p* = 0.025) that was attenuated after further adjustment for quantitative MRI scores (B = −0.21, SE = 0.12, *p* = 0.081). Similar to CDR-SB at baseline, no significant interaction between quantitative MRI scores and MBI on global cognitive z-scores was found at baseline (B = 0.34, SE = 0.39, *p* = 0.384). However, over the course of the study, MBI participants showed greater global cognitive decline (B = −0.007, SE = 0.002, *p* < 0.001), independent of quantitative MRI scores. The linear interaction between quantitative MRI scores and MBI on cognitive decline was not statistically significant (B = 0.006, SE = 0.005, *p* = 0.178). However, exploratory stratified analysis showed that MBI participants with low (B = −0.007, SE = 0.002, *p* = 0.002) but not high (B = −0.003, SE = 0.003, *p* = 0.265) quantitative MRI scores showed greater cognitive decline (Fig. 2).

### Model fit comparison

Further evaluations were performed to determine if the quantitative MRI model resulted in a better fit compared to the non-nested models of either CeVD or neurodegeneration biomarkers alone. For the association with MBI diagnosis, comparisons of BIC and interpretation of the Bayes factors indicated positive evidence that the combined quantitative MRI model resulted in a better fit compared to CeVD-alone (ΔBIC = 3.17, Bayes factor = 2–6) or neurodegeneration-alone (ΔBIC = 6.00, Bayes factor = 2–6). For interaction with MBI on the CDR-SB trajectory, the interaction model with quantitative MRI scores resulted in a better fit compared to interaction with CeVD-alone (ΔBIC = 62.36, Bayes factor > 10) or neurodegeneration-alone (ΔBIC = 153.96, Bayes factor > 10).

### Quantitative MRI scores, MBI, and progression to dementia

**Figure 3.**
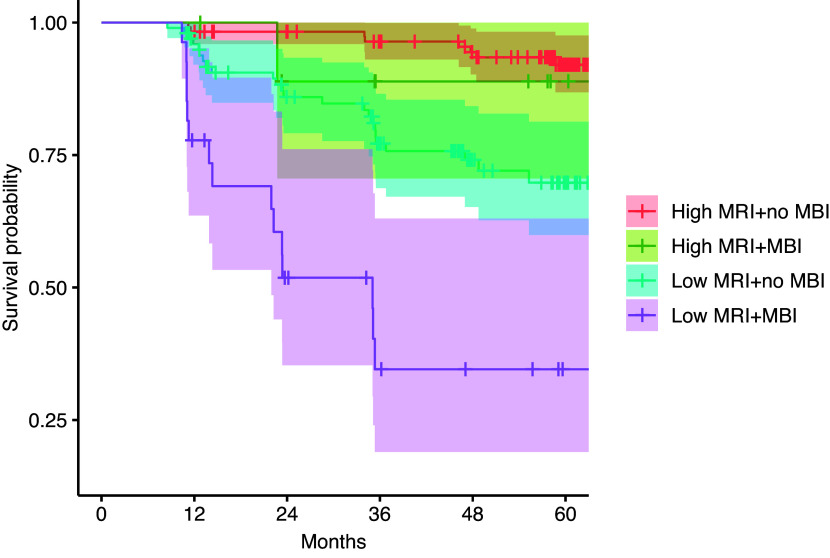
Cumulative survival rate against progression to dementia as a function of quantitative MRI score and MBI diagnosis. Stratified Cox proportional hazards models, adjusted for age, sex, education, clinical diagnosis, and TIV at baseline showed that a greater risk of progression to dementia in MBI participants with low (HR = 2.96, 95% CI = 1.54, 5.66, *p* = 0.001) but not high (HR = 1.72, 95% CI = 0.20, 14.70, *p* = 0.622) quantitative MRI scores.

16 participants with MBI (43.2%) and 33 in the non-MBI group (15.3%) developed dementia. The presence of MBI was associated with a higher risk of progression to dementia (Hazard ratio (HR) = 2.97, 95% CI = 1.61, 5.48, *p* < 0.001), independent of quantitative MRI scores and covariates. While the linear interaction between quantitative MRI scores and MBI on progression to dementia was not statistically significant (HR = 0.19, 95% CI = 0.02, 1.41, *p* = 0.103), stratified analysis showed that MBI participants had a significantly greater dementia risk in conjunction with low (HR = 2.96, 95% CI = 1.54, 5.66, *p* = 0.001) but not high (HR = 1.72, 95% CI = 0.20, 14.70, *p* = 0.622) quantitative MRI scores (Fig. 3).

## Discussion

In this MRI study of a dementia-free elderly memory clinic cohort, lower quantitative MRI scores indicating a higher burden of comorbid CeVD and neurodegeneration were associated with the presence of MBI, driven primarily by a larger WMH volume and lower cortical thickness. The composite MRI measure of mixed pathologies yielded a 3.8-fold likelihood of MBI in contrast to 1.5-fold with WMH volume or cortical thickness when considered alone. None of the individual MRI measures were associated with MBI after correction for multiple comparisons, further emphasising the utility of examining contributions of co-pathologies to MBI. In addition, MBI was associated with greater cognitive and clinical impairment at baseline and over time; participants with MBI and a higher burden of comorbid CeVD and neurodegeneration concomitantly showed the greatest cognitive and clinical decline over time, suggesting that MBI in conjunction with mixed pathologies may contribute substantially to poorer prognosis.

As a potential marker for greater cognitive decline and disease progression, MBI and dementia likely share some common pathological aetiology. Studies investigating the associations of MBI with AD pathology have reported mixed findings for brain atrophy (Matuskova *et al*., 2021; Shu *et al*., 2021), beta-amyloid burden (Lussier *et al*., 2020), and tau burden (Johansson *et al*., 2021). In our study, we found lower cortical thickness in AD-related brain regions in MBI participants, aligning with previous MBI studies showing a smaller volume in the entorhinal cortex (Matuskova *et al*., 2021) and temporal gyrus (Shu *et al*., 2021). Although hippocampal atrophy is an established MRI biomarker of memory impairment, MBI individuals in our study did not show a significantly smaller hippocampal volume compared to their non-MBI counterparts. This result suggests that the MBI syndrome may also be partially explained by non-AD pathology and is consistent with the cognitive profile of MBI involving impairment not just in memory, but also other cognitive domains such as executive function, attention, and language (Creese *et al*., 2019; Rouse *et al*., 2020; Kan *et al*., 2022a). In parallel, we found that MBI was associated with larger WMH volumes, highlighting the role of CeVD amidst equivocal findings in existing MBI literature. Our current results on the individual MRI biomarkers support MBI being reflective of a neurobehavioral risk state for all-cause dementia and is likely a multifactorial syndrome influenced by both cerebrovascular and neurodegenerative pathologies.

The prevalence of mixed AD and CeVD increases with age, accounting for most dementia cases in the community (Schneider *et al*., 2007), particularly in Asian populations (Meguro *et al*., 2012; Chen *et al*., 2016). Hence, there has been increasing interest in capturing the global pathophysiologic burden of not just CeVD (Xu *et al*., 2018; Jokinen *et al*., 2020), but also comorbid pathologies for disease prediction (Brickman *et al*., 2018; Wang *et al*., 2018). Incorporating biomarkers of CeVD (WMH volume and infarcts) and neurodegeneration (cortical thickness and hippocampal volume) into a single composite quantitative MRI measure allows for the investigation of the combined influence of underlying AD and vascular pathologies on MBI. In particular, individuals having MBI with a higher burden of comorbid CeVD and neurodegeneration showed the most pronounced objective clinical decline over time. All models involving the quantitative MRI measure in association with MBI and their interactions on clinical trajectory yielded a better fit compared to the reduced models with either CeVD or neurodegeneration alone. This suggest that determining the potential underlying neural substrates of MBI as a heterogeneous behavioural syndrome and studying their downstream clinical impact may benefit from taking a multi-lesion MRI approach that accounts for the detrimental contribution of different pathologies.

CeVD and brain atrophy also share common lifestyle-related risk factors (e.g. hypertension and diabetes mellitus) and pathophysiological mechanisms (e.g. inflammation and oxidative stress) (Santos *et al*., 2017; Wang *et al*., 2018). Neuropathological studies have also found associations of brain volumes with both AD and cerebrovascular pathologies, including neuritic plaques, neurofibrillary tangles, and brain infarcts (Erten-Lyons *et al*., 2013). Furthermore, a vicious pathological cycle in the ageing brain involving cerebral microvascular changes, pathogenic factors (e.g. impaired cerebral perfusion, neurovascular regulation, and blood-brain-barrier function), brain volume loss, and further structural cerebral lesions has also been widely discussed (Attems and Jellinger, 2014; Liu *et al*., 2015). While MRI markers for CeVD and neurodegeneration do not necessarily reflect distinct brain aetiologies, our results demonstrate that the global burden of mixed cerebrovascular and neurodegenerative brain insults may be a stronger contributor to MBI and also further exacerbate the impact of MBI on clinical trajectory than individual biomarkers. Although this study was unable to determine causality between various neuroimaging pathologies, our findings suggest a strong possibility of a synergistic contribution of cerebrovascular and degenerative factors to MBI.

Integrating multiple biomarkers of underlying mechanisms into a composite measure in conjunction with MBI evaluation may contribute to a more comprehensive risk profile of pathological burden and early neuropsychiatric manifestation of dementia. With increasing accessibility and accuracy of automated brain segmentation and lesion identification technology, a neuroimaging pipeline that generates a quantitative MRI metric could potentially be implemented into clinical evaluations in conjunction with early neuropsychiatric changes, to help identify memory clinic patients at heightened risk of cognitive decline for enrolment into clinical trials, early interventions, and clinical management. The quantitative MRI measure may also contribute to a more individualised treatment strategy for individuals with MBI or serve as a useful monitoring marker in intervention studies than single MRI biomarkers.

While the strengths of the current study include the comprehensive neuroimaging and longitudinal neuropsychiatric and neurocognitive assessments, causality between brain pathologies, MBI, and cognitive decline could not be established due to its observational nature. This study also has several other limitations. Firstly, this study has a relatively modest sample size and the use of repeated NPI assessments that were informant-based to classify MBI may have led to incomplete NPS data and a lower prevalence of MBI. This has prevented analysis on individual MBI domains and may have limited our statistical power to detect a significant linear interaction between quantitative MRI scores and MBI on cognitive trajectory and incident dementia. Future replication studies with a larger sample of MBI cases or using the more recently developed MBI Checklist (Ismail *et al.,* 2017)^12^ to better capture the symptomatology of the MBI syndrome will be needed. Secondly, our findings in memory clinic participants with high pathological burden may not be generalisable to broader, community dwelling populations globally. While significant associations of the quantitative MRI measure with cognitive impairment and NPS have been demonstrated previously in community-based populations (Tan *et al*., 2020; Kan *et al*., 2022b), our results would benefit from future investigations in more diverse cohorts. Thirdly, other potential confounders such as lifestyle factors and medication use were not accounted for in the analysis. Lastly, our analyses were restricted to MRI biomarkers of vascular and degenerative pathologies and future studies could potentially examine if the quantitative MRI measure is associated with plasma biomarkers of AD and CeVD that are more economical to assess.

In conclusion, this longitudinal study demonstrates that lower quantitative MRI scores, reflecting a higher burden of comorbid CeVD and neurodegeneration, were associated with the MBI syndrome, even in the pre-dementia stage. MBI individuals with higher burden of comorbid CeVD and neurodegeneration showed the greatest cognitive and clinical decline, suggesting that incorporating assessment for persistent neuropsychiatric disturbances with a composite MRI-based approach that accounts for multiple pathologies may help to identify individuals at the highest risk of accelerated decline for clinical management or preventative intervention.
